# Gender discrimination as a barrier to high-quality maternal and newborn health care in Nigeria: findings from a cross-sectional quality of care assessment

**DOI:** 10.1186/s12913-021-06204-x

**Published:** 2021-03-04

**Authors:** Chioma Oduenyi, Joya Banerjee, Oniyire Adetiloye, Barbara Rawlins, Ugo Okoli, Bright Orji, Emmanuel Ugwa, Gbenga Ishola, Myra Betron

**Affiliations:** 1 Maternal and Child Survival Program and Jhpiego, Abuja, Plot 971 Reuben Okoya Crescent, Wuye District, P.O. Box 14832, Abuja, FCT Nigeria; 2 Maternal and Child Survival Program and Jhpiego, 1776 Massachusetts Ave, NW, Suite 300, Washington, DC, 20036 USA

**Keywords:** Gender analysis, Gender-sensitive, Gender inequality, Inequity, Gender-based violence, Family planning, Antenatal care, Quality of care, Health workforce, Respectful maternity care

## Abstract

**Background:**

Poor reproductive, maternal, newborn, child, and adolescent health outcomes in Nigeria can be attributed to several factors, not limited to low health service coverage, a lack of quality care, and gender inequity. Providers’ gender-discriminatory attitudes, and men’s limited positive involvement correlate with poor utilization and quality of services. We conducted a study at the beginning of a large family planning (FP) and maternal, newborn, child, and adolescent health program in Kogi and Ebonyi States of Nigeria to assess whether or not gender plays a role in access to, use of, and delivery of health services.

**Methods:**

We conducted a cross-sectional, observational, baseline quality of care assessment from April–July 2016 to inform a maternal and newborn health project in health facilities in Ebonyi and Kogi States. We observed 435 antenatal care consultations and 47 births, and interviewed 138 providers about their knowledge, training, experiences, working conditions, gender-sensitive and respectful care, and workplace gender dynamics. The United States Agency for International Development’s Gender Analysis Framework was used to analyze findings.

**Results:**

Sixty percent of providers disagreed that a woman could choose a family planning method without a male partner’s involvement, and 23.2% of providers disagreed that unmarried clients should use family planning. Ninety-eight percent believed men should participate in health services, yet only 10% encouraged women to bring their partners. Harmful practices were observed in 59.6% of deliveries and disrespectful or abusive practices were observed in 34.0%. No providers offered clients information, services, or referrals for gender-based violence. Sixty-seven percent reported observing or hearing of an incident of violence against clients, and 7.9% of providers experienced violence in the workplace themselves. Over 78% of providers received no training on gender, gender-based violence, or human rights in the past 3 years.

**Conclusion:**

Addressing gender inequalities that limit women’s access, choice, agency, and autonomy in health services as a quality of care issue is critical to reducing poor health outcomes in Nigeria. Inherent gender discrimination in health service delivery reinforces the critical need for gender analysis, gender responsive approaches, values clarification, and capacity building for service providers.

## Background

Nigeria has one of the highest rates of maternal mortality in the world (576 deaths per 100,000 live births) [1] and accounts for 19% of the world’s total maternal deaths [2]. Reproductive, maternal, newborn, child, and adolescent health (RMNCAH) outcomes are poor in Nigeria due to low coverage of health services such as antenatal care (ANC), high unmet need for FP, low rates of facility-based childbirth, poor quality of services, and an array of inequities and inequalities [3–9].

Research in recent years has increasingly demonstrated that gender-based attitudes and practices of health providers and gender dynamics in health facilities contribute to issues of access and quality of RMNCAH care. Gender norms frequently expose women to early or forced marriage, adolescent pregnancies, unintended pregnancies, and sexual or physical violence [10]. These biases and norms include women’s subordinate position within the home, lack of control over household decision-making (including health-seeking decisions), lack of money to pay for transport to distant facilities, and lack of mobility outside the home without male permission or a chaperone [5, 11–13]. Where women lack autonomy and mobility outside the home, their access to safe, adequate, timely, and affordable health services, particularly emergency obstetric care, is undermined [14, 15]. These norms also influence whether or not people seek care and the quality and effectiveness of the care as the Lancet series on maternal health identified gender inequality as a barrier to accessing high-quality care, noting that gender inequality influenced women’s decision-making for seeking health care [16, 17]. Even when health services are available, gender bias and harmful norms can lead to sex-based inequities in accessing services [18].

A large FP program in six Nigerian cities (2009–2015) found that many Nigerian health providers discouraged the use of contraceptives among women who were newly married because they believed that women should have children immediately after marriage. Providers often believed that people with small families should have bigger ones or that women should obtain the consent of their husband to receive contraception [19]. In a 2018 study in South West Nigeria, providers encouraged young, sexually active, unmarried clients to abstain from sex instead of using a FP method, discouraged women from using contraceptives (due to the mistaken belief that contraceptives impair future fertility), and sometimes requested a husband’s permission before providing a woman with contraceptives [20].

Increased rates of skilled birth attendance and facility-based childbirth that meet basic quality standards are key to reducing maternal mortality and morbidity. Gender discrimination in health service delivery leads to poor quality care that can prevent women from visiting facilities even when health services are available [21]. Approaches to quality of care in low-resource settings have mainly focused on the clinical effectiveness of care. But recognition of clients’ preferences and experience of care as central elements for improving the quality of person-centered health services is increasing [22, 23]. The 2016 WHO Quality of Care framework for improving maternal and newborn health care emphasizes experience of care, which includes respectful client-provider interaction as a core dimension of quality of care and a key determinant of women’s use of services [24].

Women are more likely to be poor than men in most societies, and this status is an important driver of providers’ mistreatment of women during care, which contributes to poor quality of care and potentially reduces women’s subsequent utilization of care [9]. Mistreatment and abuse of mothers and newborns includes failure to meet professional standards of care, poor rapport between women and providers, [10], physical abuse, non-consented clinical care, non-confidential care, non-dignified care (including verbal abuse), discrimination based on specific patient attributes, abandonment or denial of care, and detention in facilities [25]. Verbal abuse, including shouting, insulting, or threatening a woman or her newborn as well as physical abuse, such as slapping a woman or her newborn, remain antiquated practices that violate the rights of patients and compromise the quality of care [26, 27]. These harmful practices reinforce gender norms and are often normalized by both providers and clients [28]. Bohren et al. conducted a study on mistreatment in childbirth in Nigeria in 2017 and found that women reported experiencing or witnessing physical abuse, including slapping, physical restraint to a delivery bed, and detainment in the hospital, and verbal abuse, such as shouting and threatening women with physical abuse. Some women were forced to give birth on the floor, unattended by a provider [21].

Women in low- and middle-income countries frequently choose not to give birth in health facilities because prior experiences of mistreatment and health facilities’ poor reputations have eroded their trust in the health system [21]. A study in Enugu State in South East Nigeria found that utilization of services is largely determined by women’s perceptions of the quality of care that will be received, specifically provider behavior [29]. Other studies in Nigeria also found that the key reasons women said they did not use facility-based maternal and child health services were poor provider attitudes [5] and perceived provider biases based on age, marital status, parity, and socio-economic status; such attitudes and biases can result in restricted services and skewed provision of information [30].

On the other hand, growing evidence suggests that positive male engagement in RMNCAH can improve access to services, quality of care, and health outcomes. The United States Agency for International Development (USAID) qualifies positive male engagement as “the involvement of men and boys across life phases in family planning, sexual and reproductive health, maternal and child health, and HIV programs as a) clients/users; b) supportive partners; and c) agents of change to improve health and gender equality outcomes, actively address power dynamics, and transform harmful masculinities. Engaging men and boys also includes broader efforts to promote equality with respect to sexual relations, caregiving, fatherhood, division of labor, and ending GBV” [31]. Increased male participation in RMNCAH that promotes couples communication, equitable joint decision-making and gender equity can also lead to greater uptake of modern FP methods, ANC services, HIV testing and treatment, facility-based childbirth, breastfeeding, housework and childcare sharing, and child immunization [32–36]. The World Health Organization (WHO) recommends the presence of a labor and birth companion of choice, if desired by a woman, as a core element of care to improve labor outcomes and women’s satisfaction with care [34, 37]. A 2013 Cochrane review found that supportive companionship increased the likelihood of vaginal births (reducing the need for cesarean sections, forceps, or vacuum delivery), reduced the need for pain medication, shortened labor, and improved newborn Apgar scores [38].

Finally, health providers also experience gender discrimination and violence that can impact the delivery of care. Violence toward health providers in their personal lives, from clients, or from others in the health workplace is not uncommon. WHO estimates that between 8 and 38% of health providers worldwide suffer physical violence at some point in their careers. Nurses are most at risk. A 2012 study on workplace violence against health providers in Abia State (adjacent to Ebonyi State) found that 88.1% of health providers had experienced workplace violence (25.1% experienced physical assault and 4.5% experienced sexual harassment) [39]. Violence not only impacts the psychological and physical well-being of health providers, but also affects job motivation and compromises the quality of care they deliver [40]. In a landscape analysis of disrespect and abuse in facility-based childbirth, Bowser and Hill noted that “the perspective of the women who provide that care, however, has remained virtually absent from the discourse” [25]. Filby et al. point to the violence and poor working conditions midwives encounter as a driver of moral distress, burnout, poor retention, and poor quality of care [41].

For efforts to improve RMNCAH outcomes in Nigeria to succeed, the impact of gender on access to care and quality of care must be understood and addressed. A gender perspective is necessary to understand health facility-level factors that deter women from seeking facility-based care [42, 43].

### Purpose of the study

Harmful gender norms can reduce women’s ability to obtain health care, influence how health providers treat women, and exclude men from reproductive health. For example, norms that dictate a woman must obtain permission to seek care for herself or children, that restrict women’s ability to make decisions about their reproductive health, or those that prevent men from participating in equitable joint-decision making around health care can lead to poor health outcomes. These norms can also subject health providers to violence and poor working conditions that, in turn, impact the quality of service delivery. Evidence shows that when clients experience poor treatment in health facilities, they are less likely to use contraceptives, deliver in a health facility, seek care for sick children, or use other health services. This perpetuates maternal and newborn death and disease.

Programs that focus on RMNCAH typically focus on women and girls. These programs may examine health conditions associated with women’s reproductive roles, but often fail to consider the unequal gender dynamics that characterize health service delivery and produce poor health outcomes for women and girls. They also often miss how women’s subordinate roles within households, communities, and societies contribute to negative health behaviors and outcomes. Additionally, programs may not consider how women’s health is impacted by the unequal division of labor, allocation of resources, decision-making, caregiving, or mobility outside the home.

This study was conducted at baseline to inform the focus and program design of an integrated maternal and child health project in Kogi and Ebonyi States supported by the USAID-funded Maternal and Child Survival Program (MCSP). MCSP in Nigeria’s goals included building the capacity of health care providers to address gender attitudes, dynamics and disparities in service delivery in the pursuit of more equitable maternal and newborn health outcomes. Among other aims, the study sought to assess whether or not gender plays a role in access to, use of, and delivery of health services— and if it does, how.

In this study, we define gender dynamics as relationships and interactions among girls, boys, women, and men. Gender-sensitivity, in this context, refers to providers’ knowledge, attitudes, practices, and beliefs about gender equity that take into account gender differences in access to health information, service delivery, and health outcomes. Providers’ knowledge of RMNCAH was assessed using structured interview guides with gender-specific questions [24]. Instances of unequal or disadvantageous treatment of clients on the basis of gender that were reported during interviews or observed during ANC consultations and L&D were considered gender discrimination.

Earlier studies recommended considering gender barriers while designing, implementing, monitoring and evaluating interventions to ensure program objectives are achieved and that efforts do not create unintended consequences, particularly for women and girls [44, 45]. Hence, a gender analysis— a systematic methodology for examining how differences in power relations result in differential risks, exposures, vulnerabilities, and outcomes in health for men and women— is required [46].

The gender analysis within this quality of care assessment sought to answer the following research questions and were categorized into the following domains from USAID’s Gender Analysis Framework [44, 46]:

1. Are gender-related factors associated with health providers’ attitudes toward clients’ access to ANC, labor and delivery (L&D), and FP services in Kogi and Ebonyi States? (Domain: Practices and participation)
2. Are health providers gender-sensitive in their attitudes and practices during ANC, L&D, and FP services? (Domains: Practices and participation, Beliefs and perceptions)
3. What gender dynamics exist among health providers in the workplace? (Domain: Institutions, laws, and policies)
4. Are there barriers to gender-sensitive maternal and newborn service delivery? (Domains: Practices and participation, Beliefs and perceptions, Access to assets)

## Methods

### Study setting and design

The baseline quality of care assessment was a cross-sectional, health facility-based study which examined service providers’ knowledge, skills, and gender-related beliefs, practices, and policies with respect to ANC services, labor and vaginal deliveries, and FP services.

Study instruments included the following: ANC Observation Checklist, L&D Observation Checklist, FP Consult Observation Checklist, and a Maternal and Newborn Health Service Provider Interview Guide and Knowledge Test for providers who offered ANC and L&D services. Clinical observations of client-provider interactions were conducted by trained, practicing clinicians who directly observed care in real-time while using structured, standardized observation checklists. The checklists were developed and used by USAID’s Maternal and Child Health Integrated program, based on WHO-recommended evidence-based practices for ANC and L&D care [47]. The structured provider interview and knowledge test was a verbally-administered, quantitative tool (vs. a self-administered survey) that primarily included close-ended questions but also a few open-ended questions on the following topics: provider background charateristics and work environment, knowledge of evidence-based maternal and newborn health interventions, experience with violent and disrespectful treatment, and gender-specific atttitudes and beliefs that can affect client care [24]. The ANC checklist and provider included questions from the Service Provision Assessment, which has been widely used in low-and middle-income countries [48].

### Sampling methodology and sample characteristics

Samples were drawn from different units of the health facilities, including the maternity, antenatal, and FP units, with clustering of data by facility. A total of 40 health facilities targeted to receive quality improvement interventions in the first phase of MCSP implementation were purposively selected from a larger list of 120 health facilities in Kogi and Ebonyi States that were identified in consultation with the State Ministries of Health to receive support from MCSP. The study was powered based on the number ANC consultations to be directly observed. For observations of labor and delivery care, the plan was to observe all deliveries during the days of the study team’s visit because of the low caseload of deliveries in most of the facilities.

Based on an assumption of 220 working days per year, ANC data extracted from registers of the selected health facilities indicated a combined average of 197 and 170 ANC visits per day in facilities in Ebonyi and Kogi, respectively. The desired sample size of ANC consultations to be observed was based on cluster sampling calculations (assuming health workers and clients are clustered within facilities) with a median design effect of 1.5 to allow + 12% precision in quality of care indicator estimates. The assumed prevalence for the quality of care indicators of interest was set at 50% to generate the most conservative sample size, with approximately 200 ANC consultations planned to be observed in each state. Target sample sizes were distributed across facility types based on identified ANC caseloads—proportional to size. Since more services took place at the tertiary level, the protocol planned for the observation of 20 ANC consultations in the tertiary facility, 12 consultations in each of the general and mission hospitals, and 5 consultations in each of the primary health centers and private clinics.

Current national standards require that a minimum of four service providers work in the maternity unit of a facility to operate a shift-duty system. Therefore, based on an estimated minimum population of 160 eligible service providers (4 providers in each of the 40 health facilities), a 5% margin of error, and a 95% confidence interval, we planned to interview 136 ANC and labor and delivery providers.

### Data collection procedures

Twenty-two obstetricians, pediatricians, medical officers, nurses, and midwives were selected as data collectors for all the study tools based on their active clinical practice and data collection experience. All data collectors received 2 weeks of training that included a briefing on the background and rationale of the study, an overview of the study instruments and informed consent process, and orientation on all data collection tools, including gender-related aspects of the observational and interview tools and technical instructions for using CommCare technology, the mobile software used for data collection. Data collectors were trained on gender terms and to review records for missing or inconsistent answers before submission. Data collectors practiced using the study instruments in the classroom with colleagues during role plays and clinical simulations using anatomic models and inter-rater reliability of the observers’ scores was tested. Field tests using the tools were conducted over 2 days in five health facilities in Kogi States, and feedback was used to revise the tools and reword questions as necessary.

Data collectors worked in teams whose staffing was based on the number of observations to be made and classifications of the health facilities. Data collection lasted 1–2 days in primary health centers and 2–4 days in larger secondary and tertiary health facilities. Repeat visits were made to complete the target number of ANC observations if needed; repeat visits were required more frequently in tertiary health facilities and general hospitals. Supervisors visited data collection teams to provide ongoing quality control.

Data were collected in Kogi and Ebonyi States from 1 April through 30 June 2016 and entered directly on android-enabled tablet PCs using custom-created data entry programs developed with the password-protected CommCare software package. Technical and information technology staff monitored data sent to the CommCare HQ online site and verified data completeness and accuracy.

### Data analysis

Data were exported from CommCare to Excel before being converted to SPSS for cleaning and analysis. Data analyses performed included percent distributions, counts, means, medians and cross-tabulations. Responses to open-ended questions from the provider interview were collated and summarized by theme. Results for Kogi and Ebonyi States were analyzed separately due to significant sociocultural and normative differences in gender and health practices. For example, 74.2% of women in Ebonyi State have undergone female genital mutilation compared to 1.7% of women in Kogi State [1].

Descriptive gender analysis was used to answer the gender assessment questions of the quality of care findings. Gender analysis emphasizes the importance of examining not only supply-side issues in health service provision, but also demand-side issues and the interrelation of the two [42]. Gender analysis can reveal the complex interplay of gender inequality and other inequities that constitute barriers or facilitators for access to health services and provider-client interactions. It can also provide baseline information about providers’ knowledge, attitudes, and practices around gender during RMNCAH service delivery and uncovered gender-related barriers that hinder the provision of quality, respectful, and equally accessible health care.

**Fig. 1:**
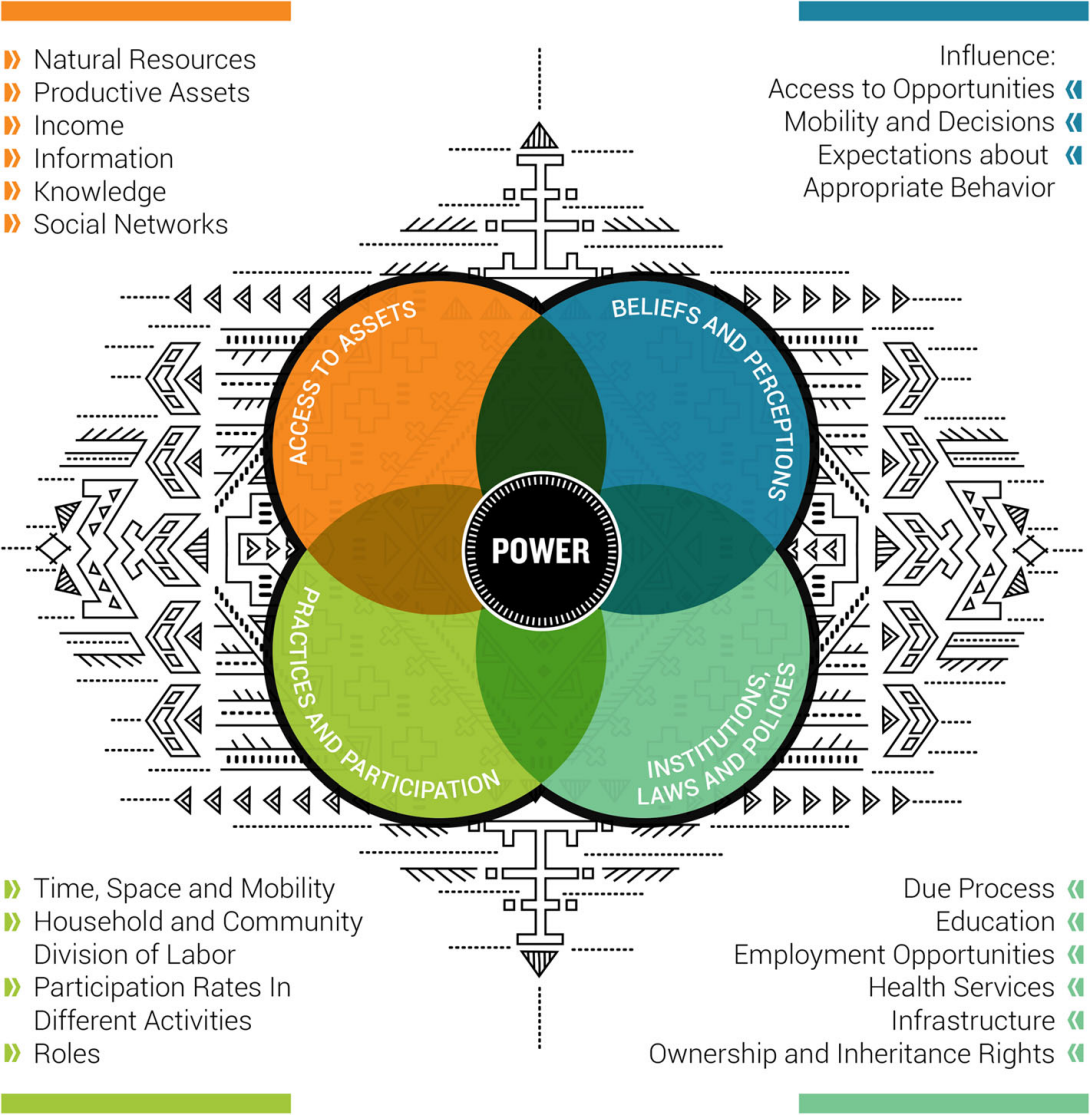
Gender analysis framework

A descriptive analysis of gender-specific quality of care findings was conducted using USAID’s Gender Analysis Framework (Fig. 1) [44, 46] to examine gender-based constraints and opportunities in four domains: (1) Practices, roles, and participation; (2) Beliefs and perceptions; (3) Access to assets; and (4) Institutions, laws, and policies:

## Results

### Sample characteristics

Twenty-six facilities were hospitals (tertiary, secondary, mission or private) and 14 were lower level clinics/health centers. (Table 1).

**Table 1:** Number of facilities assessed by type

Facility type	Ebonyi State	Kogi State	Total
Tertiary	1	1	**2**
Secondary	10	10	**20**
Mission	2	2	**4**
Private	2	2	**4**
Primary health center	5	5	**10**
Total	20	20	**40**

Although the majority of the providers were female (73%), 40% of the supervisors were male. Most providers were between the ages of 30–59 as shown in Table 2.

**Table 2:** Providers Characteristics

		Ebonyi State (***n*** = 72)	Kogi State (***n*** = 67)	Total (***n*** = 138)
		%		
Sex of supervisor	Male	44.6	35.2	40.0
	Female	55.4	59.3	57.3
	Not stated	0.0	5.6	2.7
Sex of health worker	Male	31.0	20.9	26.1
	Female	69.0	79.1	73.9
Age of health worker	20–29 years	2.9	7.1	4.8
	30–39 years	22.9	14.3	19.0
	40–49 years	54.3	32.1	44.4
	50–59 years	17.1	42.9	28.6
	Not Stated	2.9	3.6	3.2
		**# of years**		
Mean age		42.82	45.70	44.10
Mean number of years holding the qualification of this post		15.91	21.93	18.53
Mean number of years working in this facility		7.20	12.41	9.47

Overall, (Table 3) 435 ANC consultations (190 in Ebonyi State and 233 in Kogi State), 47 L&Ds (19 in Ebonyi and 28 in Kogi) were conducted. Additionally, 138 maternal and newborn health providers (71 in Ebonyi and 67 in Kogi) were interviewed about maternal and newborn health topics, as well as their knowledge, beliefs, and perceptions about gender and workplace gender dynamics. Providers included community health extension workers, midwives, nurses, nurse-midwives, general doctors, obstetricians, pediatricians, and other specialists who offered ANC and/or L&D services.

**Table 3:** Number of ANC and L&D observations, and provider interviews

	Ebonyi State	Kogi State	Total
# ANC consultations observed	202	233	435
# L&D observed	19	28	47
# Providers interviewed	71	67	138

### Findings

The results of the assessment are categorized according to the gender analysis framework domains that relate to quality of care most strongly: Beliefs and perceptions; Practices, roles, and participation; and Institutions, laws, and policies.

#### Beliefs and perceptions

This domain includes gendered norms, such as attitudes and beliefs about what it means to be a woman or a man in a specific context. Beliefs and perceptions affect a person’s behavior, participation, dress, and decision-making capacity [10].

Observation of ANC consultations and interviews with providers revealed that gender-inequitable attitudes toward service provision are prevalent where 98% of providers agreed that men should be involved in RMNCAH services, but only 10% asked women if they wanted their partner to participate in ANC. Providers had deep-rooted patriarchal beliefs and perceptions about gender, women’s autonomy, and gender-based violence (GBV) hence information on gender-based violence (GBV) or referrals to GBV services were not offered at all. Providers held contradictory beliefs that women were responsible for pregnancy, childbirth, and childcare, but that men should be the primary decision-makers controlling whether women seek care, including whether or not to use contraceptives as only about 3% of pregnant women were asked about who the decision maker will be for labour and delivery. The study also showed an acceptance by providers and clients of practices related to mistreatment of women and their newborns during facility-based care as about 51.6% providers engaged in at least one harmful practice during labour and delivery (Table 5).

Ninety-five percent of providers agreed or strongly agreed that every woman who visits the facility should be given the same quality of treatment irrespective of whether she has a companion. Still, 4.2% of providers disagreed, strongly disagreed or were neutral that women without accompanying partners should be treated the same way as any other patient.

In Ebonyi State, 67.6% of healthcare providers disagreed or strongly disagreed that a woman should be able to choose a FP method on her own, compared to 50.7% of providers in Kogi State. Providers also held moralistic beliefs about contraceptives and premarital sex. In both states combined, 23.2% of providers disagreed that unmarried clients should use FP.

#### Practices, roles, and participation

This domain includes roles and responsibilities that are traditionally expected of men and women, which are influenced by gender norms and beliefs [10]. The majority of gendered practices related to patient-provider interactions and how patients were treated by providers. Other issues concerned experiences of violence by both clients and health providers. Across both districts, the majority of health providers strongly agreed (73.2%) or agreed (26.8%) with the statement: “Both male and female clients deserve to receive services without violence.”

The majority of ANC providers observed greeted clients in a friendly and respectful manner (Table 4). However, few providers asked clients if they would like their husband/partner to participate in ANC consultation.

**Table 4:** Observations of provider interactions with clients during ANC counseling

Checklist Component	Ebonyi State % (***n*** = 202)	Kogi State % (***n*** = 233)	Total % (***n*** = 435)
Provider greeted the client and others present in a friendly and respectful manner	91.6	89.3	90.3
Provider introduced him/herself and gave his/her title	5.9	17.2	12.0
Provider called the client by her appropriate name/title	70.8	56.7	63.2
Provider informed client about progress of the pregnancy	42.6	47.2	45.1
Provider asked the client where she will deliver	10.4	6.0	8.1
Provider asked the woman if she wanted her husband/partner to participate in ANC consultation	7.9	12.4	10.3
Provider asked client if she has identified a birth companion of her choice	1.5	1.7	1.6

Respectful maternity care findings for women in labor were mixed. During the initial client assessments for women in labor, the majority of clients (90%) were respectfully greeted by providers. However, only 45% of providers encouraged women to have a support person present during labor and birth, and only 50% of providers asked women (and the support person, if present) if they had any questions. Notably, no providers in either state told the woman or her companion what was going to be done, listened to the woman or provided support and reassurance.

During L&D, providers made an effort to provide respectful care. More than half of providers (57.1% in Ebonyi State and 76.9% in Kogi State) explained the procedures being performed to women. However, a gap in quality of services related to vaginal examinations was identified between the two states. In Ebonyi State, 28.6% of clients were informed before a vaginal examination was conducted compared with 92.3% of clients in Kogi State. Similarly, only 14.3% of clients in Ebonyi State were informed of the examination findings compared with 92.3% of clients in Kogi State.

At least one potentially harmful practice, such as applying fundal pressure to hasten delivery of baby or placenta, was performed during delivery in 59.6% of encounters and at least one disrespectful or abusive practice was observed in 34.0% of encounters across Ebonyi State and Kogi States (Table 5). Episiotomies were performed in at least one-quarter of the observations across the two states.

**Table 5:** Potentially harmful practices observed during delivery

	Ebonyi State % (***n*** = 19)	Kogi State % (***n*** = 28)	Total % (***n*** = 47)
**Engaged in at least one harmful practice**	**63.2**	**57.1**	**59.6**
**Potentially harmful practices**			
Used an enema	0.0	3.6	2.1
Applied fundal pressure to hasten delivery of baby or placenta	10.5	10.7	10.6
Performed lavage of uterus after delivery	0.0	7.1	4.3
Stretched the perineum	0.0	21.4	12.8
Manually explored the uterus after delivery	15.8	21.4	19.1
Performed episiotomy	6.4	2.1	8.5
Performed routine aspiration of newborn mouth and nose at birth	15.8	35.7	27.7
Started routine intravenous line without indication	10.5	0.0	4.3
Restricted food and fluids in labor	31.6	32.1	31.9

#### Institutions, laws, and policies

This domain includes the ways in which women and men are dissimilarly affected by institutional structures, policies, and rules both within the health system and beyond and includes considerations of formal and informal rights [10]. Violence directed toward health providers is included within this domain as it occurs at the institutional level, must be addressed at the institutional level, and can affect the care patients receive.

Seventy-eight percent of providers had received no training on gender, gender-based violence or human rights in the last 3 years. Fewer than half of providers in Ebonyi (40.8%) and Kogi (31.3%) reported that their facilities were equipped to allow for the presence of a birth companion through ensuring visual privacy in the delivery ward. Most facilities were open wards where multiple women delivered without a wall, curtain or other visual barrier. As a result, men who accompanied their partners for L&D were often not allowed (according to the facility’s policy) inside the labor or postnatal wards to act as supportive companions. The majority of providers did not allow women to choose their delivery position; supine, dorsal, or lithotomy positions were permitted, but women were unable to deliver in a non-horizontal position.

**Fig. 2:**
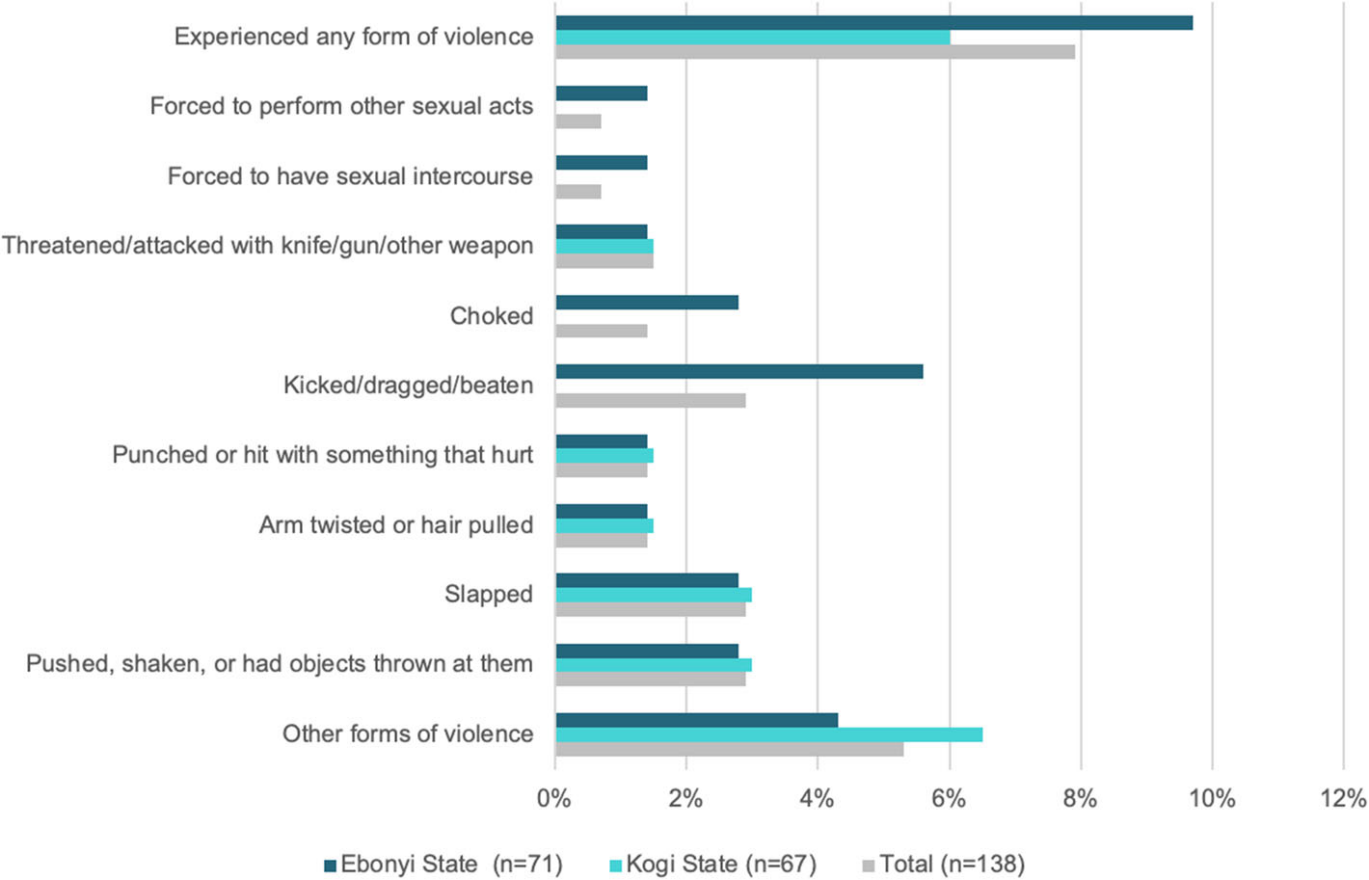
Provider-reported incidence of violence against themselves or other providers in the workplace

While the majority of providers interviewed believed that they were treated respectfully in the facility, 8% of providers across the two states reported that they or a colleague had experienced at least one form of violence by a colleague or supervisor (Fig. 2). Violence in the workplace was more frequently reported among health providers in Ebonyi state (9.7%) than Kogi state (7.9%). No experiences of sexual violence were reported in Kogi State, but 1.4% of the female health providers in Ebonyi State reported being physically forced to have sexual intercourse or perform other sexual acts while on the job. Providers were not asked about whether the violence was perpetrated by co-workers, supervisors or clients. Physical violence was reported to occur more frequently in the workplace in Ebonyi State (7.9%) than in Kogi State (1.4%).

**Fig. 3:**
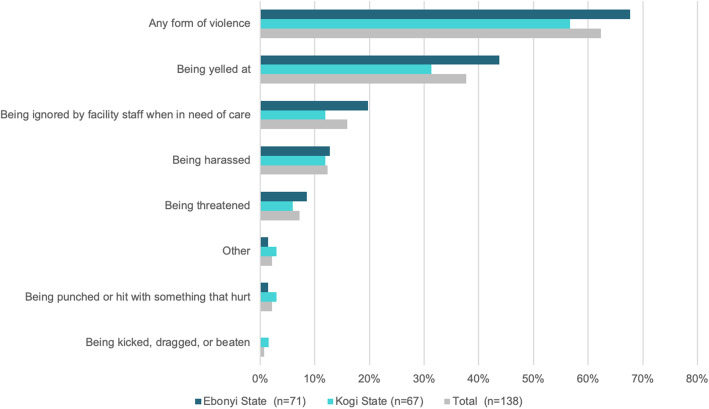
Provider-reported Incidence of violence against clients in the health facility by providers

Sixty-seven percent of providers also reported high rates of experiencing, observing or hearing of at least one incident of violence against clients (Fig. 3).

## Discussion

Gender, age, and marital status should not affect the right to receive high-quality, gender-sensitive, and respectful services when seeking ANC and L&D care or other health services, such as family planning. Yet gender norms embedded in sociocultural practices persist, and drive providers’ poor attitudes, perpetuate violence, limit the utilization of facility-based services, and contribute to poor RMNCAH outcomes [5, 6]. It is worth noting that age and sex clearly did not show any remarkable difference throughout the study as beliefs and practices seem to cut across age and sex of female providers and the male providers and between older and younger providers as 73.9% of providers interviewed were women while over 70% were aged 40-59 years. Apparently the belief systems and practices found in the study indicates social acceptance and cuts across the two genders (male and female). The current findings have implications for designing interventions to help improve the provision of gender-sensitive and respective care: program planners must be intentional about addressing and measuring inequalities, as well as improving quality, respectful care.

### Beliefs and perceptions

Virtually all healthcare providers surveyed in both states (98.5%) agreed that men play a role in maternal, newborn, and child health.. This is consistent with previous findings from hospitals in Nigeria where midwives acknowledged the benefits of having a partner present, for example, contributing to pain relief during childbirth [49]. Previous studies have found that engaging men in reproductive, maternal, and newborn health can increase care seeking, improve home care practices, and support more equitable communication and decision-making among couples related to maternal and newborn health [1, 36]. Despite this recognition, facilities did not have adequate privacy in the L&D and postpartum wards to enable men to attend L&D and did not allow or encourage men to participate. At the same time, the finding could imply that many providers believe the man should be the decision-maker about a woman’s reproductive health, given that providers’ subsequent responses prioritized men’s decision-making authority over women’s reproductive autonomy.

However, as a reflection of gender norms that prioritize men’s power in decision-making, most providers did not think women should have autonomy in FP decision-making—67.6% of providers interviewed in Ebonyi State and 50.7% in Kogi State believed that a woman should not choose a FP method on her own. Even though multiple studies have shown FP to be generally accepted as women’s responsibility [50], in Kogi and Ebonyi States, providers believed the decision of whether or not to use FP should be made by the man or by the couple together, and the woman should be responsible for implementing FP decisions. A previous study in Nigeria found that men often think that women should take responsibility for using contraception, but that men should control the decision-making [51]. These perspectives may be at odds with current programs in Nigeria that direct FP awareness raising toward women alone, excluding men, given that Nigerian couples often do not discuss FP [52] and that men typically do not participate in FP consultations.

Providers also held discriminatory beliefs about who should be allowed to use FP. Beliefs were based on culture, gender, and religion rather than medical need or client preference. According to the Demographic and Health Survey, “Women and men in Nigeria tend to initiate sexual activity before marriage.” Approximately one-third of women in Ebonyi and in Kogi had sex before the age of 18, but the median age of marriage for women in Nigeria was 18.1 [1]. Our study found that 23.2% of providers did not think unmarried clients should use FP services. A study in Ibadan, Oyo State, Nigeria, found that 57.5% of providers believed that unmarried adolescents should be told to abstain from sex rather than be provided with contraceptives, which they believed would promote sexual promiscuity. Providers also believed that contraceptives should not be provided to adolescents, whether married or unmarried [53]. Another program in Nigeria found that providers turned away unmarried clients, newly married couples, or couples with only one baby from FP services based on personal beliefs that unmarried clients should not be having sex and that newly married couples should begin childbearing right away to produce large families [19].

### Practices, roles, and participation

As in many health settings globally, we found that the majority of health providers were female, but the majority of supervisors were male [10, 41]. This relative exclusion of women from equitable leadership positions could be due to a number of factors, including discriminatory attitudes about women’s ability to be managers, a lack of gender-sensitive workplace policies such as breastfeeding rooms and parental leave, and sexual harassment and violence. These factors have been shown to lead to burnout, attrition, mistreatment of patients, and the delivery of poor quality health services [10].

Birth preparedness counseling observed during ANC consultations revealed low levels of interaction and engagement between providers and clients. Women were inadequately informed about the status of their pregnancy and their options for childbirth, which may reflect providers’ bias about women’s agency and dignity.

Over one-third of respondents reported having experienced, observed, or heard of at least one incident of violence or mistreatment against clients. This included being yelled at, threatened, or ignored by facility staff and, in a minority of cases, being punched, kicked, dragged, or beaten.

Mistreatment of women in labor is common in many RMNCAH service delivery settings [6]. Our study observed no occurrence of slapping, hitting, or pinching clients during or after labor in either state. However, potentially harmful practices were observed. For example, routine episiotomies that are not required (and put women at risk of harm, infection, and sepsis) signify acts of mistreatment [54]. Our findings are consistent with an earlier study that found women’s perception of quality of care was lowest related to respect for clients [55].

### Institutions, laws, and policies

Enhancing privacy during care was a gender-based constraint to accessing high-quality RMNCAH care. Our study found that only 36% of facilities were equipped to accommodate male birth companions due to limited privacy. Despite the recognition that engaging men in maternal and newborn health is beneficial [1, 36], even if men wanted to accompany their wives, facilities were unequipped to allow men to do so while maintaining the privacy of other clients.

### Study strengths and limitations

This was a small-scale cross-sectional study that included direct observation of antenatal and labor and delivery care, the gold standard for understanding quality of care; and interviews with health care providers to inform programmatic activities that strengthen the quality of RMNCAH service delivery. Observations were limited to ANC consultations and and births that occurred on the days data collectors were present. The final number of L&D observations was small due to low caseloads therefore the margins of error are wide. However, the study was not designed to be representative of the entire country but to provide baseline data within the two states to inform local project design. Another limitation is that it was unfeasible in this study to track specific providers’ knowledge, attitude and practives (KAPs). Also the sex and age of providers were not specifically compared with their beliefs and practices. Given that health service providers across Nigeria operate under similar conditions and that the gender norms present in our study exist throughout Nigeria, we believe that the findings of this gender analysis can effectively inform gender integration for maternal and newborn health programming across the country.

Providers may have delivered care differently because they were under observation (Hawthorne effect), resulting in underreporting of gender discrimination or mistreatment in care. Social desirability bias may have impacted providers’ interview responses.

Another limitation of the study stems from the sensitivity towards terms such as *gender*, *gender-based violence, disrespect and abuse*, or *mistreatment* among providers in Nigeria. These terms were included in the survey instruments and potentially affected responses from providers because these terms may have elicited negative reactions, particularly for questions regarding workplace gender dynamics. Widespread conflation of the term “gender” with women’s issues—which are often dismissed as a western imposition, a modern fad, an attack on men’s rights, an attack on tradition/culture/religion, or an accusation that all men are bad—may have influenced respondents interpretations of the term. Some respondents may have not understood what was meant by gender within the study. Additionally, some types of violence may not have been considered violent by respondents due to the high acceptance of violence against women and the culture of silence surrounding gender-based violence in Nigerian society. Further validation of the study tools would have helped to limit misinterpretation.

## Recommendations

Gender-discriminatory beliefs and practices identified in our study hold far-reaching implications for the ability of women to make self-directed decisions about RMNCAH. Gender-discrimination negatively impacts the ability of providers to deliver gender-sensitive care that respects women’s human rights, dignity, and bodily autonomy [19]. For RMNCAH programming in Nigeria to be successful, programs must meaningfully engage men, women, and community leaders in awareness raising, in ways that respect women’s reproductive autonomy, agency and rights. And efforts must go beyond just the benefits of healthy timing and spacing of pregnancies and limiting family size. Capacity building of providers, as well as health facility’s and national policies, should reinforce that health service delivery should not be influenced by morals, gender biases, or religion, but should focus on medical needs, client preferences, and evidence-based approaches to care.

Our findings indicate an opportunity to improve reproductive health outcomes and leverage couples counseling to mitigate power imbalances between men and women around fertility and encourage women to participate in joint decision-making. In order to transform perceptions of RMNCAH services from being solely a woman’s issue to a joint endeavor between couples [23], previous studies [16, 56] recommended the creation of a supportive and male-friendly environment at health facilities that encourages men to be involved in maternal health services [22]. Further interventions are therefore needed at the institutional level to ensure that men are able to accompany their partners to L&D, including creating private L&D and postpartum spaces within health facilities, sensitization, training and guidance for health providers on how to engage men along the RMNCH continuum.

Such capacity building, guided by a 2018 gender capacity building framework for providers [57], can improve providers’ ability to counsel men and couples and advocate for facility preparedness to engage men in pregnancy and childbirth (when women desire men’s presence). Such efforts, however, must ensure that attempts to engage men do not infringe upon women’s reproductive autonomy by encouraging men to take control of reproductive health decision-making. Instead, they should increase and uphold women’s agency, self-efficacy, and decision-making power.

Health providers were identified as having a key role in changing the negative effects of harmful gender norms and stereotypes by empowering both women and men to make informed choices about their health. A study on improving reproductive health outcomes, Stover et al. highlighted the importance of creating opportunities for providers to clarify personal values and offer services in a nonjudgmental way to meet clients’ reproductive health needs [58].

There are not many RMNCAH interventions which address gender as a determinant of mistreatment during maternal and newborn health care [10]. Interventions include provider trainings to clarify values and transform attitudes in order to facilitate understanding of gender-discriminatory behaviors and attitudes, which influence mistreatment during labor and childbirth (for example, the WHO Health Workers for Change quality of care curriculum [59] and the Jhpiego Gender Transformation for Health Toolkit) [60]. These can be part of wider efforts to engage policymakers to focus on mistreatment during labor and childbirth and to support accountability by strengthening community and health facility linkages, putting in place systems to gather patient complaints and feedback and developing patient charters at the facility level [10]. Interventions that support a positive work environment for health providers are also needed. For example, the Heshima Project in Kenya worked at the community, facility and policy levels to examine the extent and causes of mistreatment in care in Kenya, and designed and implemented interventions to promote respectful care [61]. MCSP provided recommendations to the Nigerian MOH including a scale up of the Health Workers for Change Curriculum; capacity building and ongoing mentorship on gender-sensitive service delivery, male engagement and couples’ counseling; and first-line support to survivors of GBV. MCSP also recommended a scale up of efforts to improve infrastructure for privacy in L&D and post-natal wards in health facilities.

## Conclusion

Our study identified several RMNCAH quality of care issues affected by gender inequalities and harmful norms in Kogi and Ebonyi States. We found that some providers upheld harmful, traditional gender norms that did not respect women’s right to make decisions about the use of contraceptives or health services. ANC providers did not offer services to survivors of sexual assault or intimate partner violence or encourage men to participate in health care for themselves, their partners, or their families. Some health providers who were observed mistreating clients and their newborns reported they were subject to disrespect and abuse themselves, including experiencing workplace physical and sexual violence. These findings point to the need to train providers and address attitudes and conditions within the health system that perpetuate gender discrimination and discourage women and men from seeking and using potentially life-saving care. Also, these findings can inform the development of gender-transformative interventions and measurement approaches to address and assess the impact of harmful gender norms and practices, as well as power imbalances between men and women, on service delivery. Integrating gender into the design of interventions and capacity building efforts is key to improving quality of services. Gender analysis remains a critical step in identifying gender-based constraints and opportunities. Empowering women, involving men, transforming service providers’ negative attitudes, and encouraging respectful care are critical approaches to promote better utilization and quality of maternal health services and, ultimately, to improve maternal and newborn health outcomes [32, 62, 63]. By identifying and addressing the influences and unintended consequences of gender discrimination in health service delivery, providers, facility managers, and stakeholders in health systems can improve countries’ progress toward universal health coverage and the attainment of national and global goals such as the Sustainable Development Goals.

## Data Availability

The de-identified datasets generated and analysed during the current study are available in USAID’s public data development library at this link: https://data.usaid.gov/Maternal-and-Child-Health/Maternal-Child-Survival-Program-Baseline-Quality-o/3zqw-f3e4

## Abbreviations

ANC: Antenatal care
DHS: Demographic and Health Survey
FP: Family planning
GBV: Gender-Based Violence
HIV: Human immunodeficiency virus
IRB: Institutional Review Board
L&D: Labor and Delivery
MCSP: Maternal and Child Survival Program
RMNCAH: Reproductive, maternal, newborn, child and adolescent health
USAID: United States Agency for International Development
WHO: World Health Organization
